# Supplementary material for: Pyruvate Dehydrogenase Kinase 1 inhibition mediated oxidative phosphorylation enhancement in cartilage promotes osteoarthritis progression
Source: BMC Musculoskelet Disord. 2023 Jul 20;24:597. doi: 10.1186/s12891-023-06585-6 (PMC10357736; doi:10.1186/s12891-023-06585-6)
Supplement: Supplementary file 1 — Additional file 1. [file 12891_2023_6585_MOESM1_ESM.zip › KEGG.Gsea.1653622432464/KEGG_BASE_EXCISION_REPAIR.html]

Details for gene set KEGG\_BASE\_EXCISION\_REPAIR[GSEA]

|  || Dataset | 55457\_collapsed\_to\_symbols.cls.cls #OA\_versus\_normal.cls.cls #OA\_versus\_normal\_repos |
| Phenotype | cls.cls#OA\_versus\_normal\_repos |
| Upregulated in class | OA |
| GeneSet | KEGG\_BASE\_EXCISION\_REPAIR |
| Enrichment Score (ES) | 0.39032316 |
| Normalized Enrichment Score (NES) | 1.4351721 |
| Nominal p-value | 0.05596107 |
| FDR q-value | 0.09420812 |
| FWER p-Value | 0.923 |
Table: GSEA Results Summary

  

Fig 1: Enrichment plot: KEGG\_BASE\_EXCISION\_REPAIR      
 Profile of the Running ES Score & Positions of GeneSet Members on the Rank Ordered List

  

| SYMBOL | TITLE | RANK IN GENE LIST | RANK METRIC SCORE | RUNNING ES | CORE ENRICHMENT || 1 | POLB | DNA polymerase beta [Source:HGNC Symbol;Acc:HGNC:9174] | 93 | 0.923 | 0.0871 | Yes |
| 2 | MPG | N-methylpurine DNA glycosylase [Source:HGNC Symbol;Acc:HGNC:7211] | 277 | 0.768 | 0.1512 | Yes |
| 3 | OGG1 | 8-oxoguanine DNA glycosylase [Source:HGNC Symbol;Acc:HGNC:8125] | 453 | 0.681 | 0.2072 | Yes |
| 4 | PARP2 | poly(ADP-ribose) polymerase 2 [Source:HGNC Symbol;Acc:HGNC:272] | 758 | 0.579 | 0.2427 | Yes |
| 5 | POLD1 | "DNA polymerase delta 1, catalytic subunit [Source:HGNC Symbol;Acc:HGNC:9175]" | 811 | 0.566 | 0.2965 | Yes |
| 6 | POLD4 | "DNA polymerase delta 4, accessory subunit [Source:HGNC Symbol;Acc:HGNC:14106]" | 820 | 0.564 | 0.3535 | Yes |
| 7 | SMUG1 | single-strand-selective monofunctional uracil-DNA glycosylase 1 [Source:HGNC Symbol;Acc:HGNC:17148] | 1068 | 0.501 | 0.3854 | Yes |
| 8 | PCNA | proliferating cell nuclear antigen [Source:HGNC Symbol;Acc:HGNC:8729] | 1557 | 0.419 | 0.3903 | Yes |
| 9 | XRCC1 | X-ray repair cross complementing 1 [Source:HGNC Symbol;Acc:HGNC:12828] | 2182 | 0.340 | 0.3766 | No |
| 10 | LIG3 | DNA ligase 3 [Source:HGNC Symbol;Acc:HGNC:6600] | 2898 | 0.261 | 0.3477 | No |
| 11 | NTHL1 | nth like DNA glycosylase 1 [Source:HGNC Symbol;Acc:HGNC:8028] | 3219 | 0.228 | 0.3462 | No |
| 12 | PARP1 | poly(ADP-ribose) polymerase 1 [Source:HGNC Symbol;Acc:HGNC:270] | 3410 | 0.210 | 0.3528 | No |
| 13 | POLE3 | "DNA polymerase epsilon 3, accessory subunit [Source:HGNC Symbol;Acc:HGNC:13546]" | 3934 | 0.162 | 0.3287 | No |
| 14 | NEIL3 | nei like DNA glycosylase 3 [Source:HGNC Symbol;Acc:HGNC:24573] | 4006 | 0.156 | 0.3391 | No |
| 15 | POLE2 | "DNA polymerase epsilon 2, accessory subunit [Source:HGNC Symbol;Acc:HGNC:9178]" | 4237 | 0.134 | 0.3349 | No |
| 16 | APEX2 | apurinic/apyrimidinic endodeoxyribonuclease 2 [Source:HGNC Symbol;Acc:HGNC:17889] | 4551 | 0.109 | 0.3217 | No |
| 17 | FEN1 | flap structure-specific endonuclease 1 [Source:HGNC Symbol;Acc:HGNC:3650] | 5212 | 0.056 | 0.2761 | No |
| 18 | MUTYH | mutY DNA glycosylase [Source:HGNC Symbol;Acc:HGNC:7527] | 5252 | 0.052 | 0.2783 | No |
| 19 | PARP4 | poly(ADP-ribose) polymerase family member 4 [Source:HGNC Symbol;Acc:HGNC:271] | 5568 | 0.027 | 0.2566 | No |
| 20 | UNG | uracil DNA glycosylase [Source:HGNC Symbol;Acc:HGNC:12572] | 6078 | -0.011 | 0.2181 | No |
| 21 | POLL | DNA polymerase lambda [Source:HGNC Symbol;Acc:HGNC:9184] | 6214 | -0.022 | 0.2099 | No |
| 22 | POLD2 | "DNA polymerase delta 2, accessory subunit [Source:HGNC Symbol;Acc:HGNC:9176]" | 6311 | -0.028 | 0.2053 | No |
| 23 | LIG1 | DNA ligase 1 [Source:HGNC Symbol;Acc:HGNC:6598] | 6944 | -0.075 | 0.1639 | No |
| 24 | POLD3 | "DNA polymerase delta 3, accessory subunit [Source:HGNC Symbol;Acc:HGNC:20932]" | 7568 | -0.125 | 0.1282 | No |
| 25 | APEX1 | apurinic/apyrimidinic endodeoxyribonuclease 1 [Source:HGNC Symbol;Acc:HGNC:587] | 8767 | -0.213 | 0.0569 | No |
| 26 | PARP3 | poly(ADP-ribose) polymerase family member 3 [Source:HGNC Symbol;Acc:HGNC:273] | 9186 | -0.246 | 0.0496 | No |
| 27 | TDG | thymine DNA glycosylase [Source:HGNC Symbol;Acc:HGNC:11700] | 10728 | -0.396 | -0.0296 | No |
| 28 | NEIL1 | nei like DNA glycosylase 1 [Source:HGNC Symbol;Acc:HGNC:18448] | 10963 | -0.425 | -0.0044 | No |
| 29 | POLE | "DNA polymerase epsilon, catalytic subunit [Source:HGNC Symbol;Acc:HGNC:9177]" | 11026 | -0.433 | 0.0351 | No |
| 30 | MBD4 | "methyl-CpG binding domain 4, DNA glycosylase [Source:HGNC Symbol;Acc:HGNC:6919]" | 11114 | -0.445 | 0.0738 | No |
| 31 | HMGB1 | high mobility group box 1 [Source:HGNC Symbol;Acc:HGNC:4983] | 12170 | -0.636 | 0.0567 | No |
Table: GSEA details [plain text format]

  

Fig 2: KEGG\_BASE\_EXCISION\_REPAIR      
 Blue-Pink O' Gram in the Space of the Analyzed GeneSet

  

Fig 3: KEGG\_BASE\_EXCISION\_REPAIR: Random ES distribution      
 Gene set null distribution of ES for **KEGG\_BASE\_EXCISION\_REPAIR**
